# Clinical and genetic investigations of five Chinese families with Birt–Hogg–Dubé syndrome: a long-term follow-up study

**DOI:** 10.3389/fmed.2025.1613154

**Published:** 2025-09-08

**Authors:** Xi Kang, Ting Guo, Ali Basit, Lv Liu, Hong Luo

**Affiliations:** ^1^Department of Pulmonary and Critical Care Medicine, The Second Xiangya Hospital, Central South University, Changsha, China; ^2^Research Unit of Respiratory Disease, Central South University, Changsha, China; ^3^Clinical Medical Research Center for Pulmonary and Critical Care Medicine in Hunan Province, Changsha, China; ^4^Diagnosis and Treatment Center of Respiratory Disease in Hunan Province, Changsha, China

**Keywords:** Birt–Hogg–Dubé syndrome, pulmonary cysts, pneumothorax, fibrofolliculomas, renal cell carcinoma

## Abstract

Birt–Hogg–Dubé syndrome (BHDS), an autosomal dominant disease, is caused by germline mutations in the folliculin (*FLCN*, NM_144997) gene. This rare disorder is characterized by a clinical triad, which includes fibrofolliculomas (FFs), renal cell carcinoma (RCC), and pulmonary manifestations such as multiple pulmonary cysts (PCs) and pneumothorax. To investigate the clinical features and genetic mutations of five unrelated BHDS families in a long-term follow-up study at the Second Xiangya Hospital of Central South University, five families and their affected patients, who met the clinical and histological criteria for BHDS and were confirmed to have *FLCN* germline mutations, were evaluated. All participants underwent a comprehensive physical examination along with other relevant tests. Three novel mutations (c.246C > A, c.625_626insAGGCAGAGCAGTTTGGAT, and c.1542_1542delA) and one previously reported mutation (c.1429C > T) in the *FLCN* gene were identified. These mutations are predicted to cause truncation of the folliculin protein, likely resulting in decreased folliculin expression. Our study expands the genetic landscape associated with BHDS and provides valuable insights for future genetic counseling and the clinical management of individuals with BHDS.

## 1 Introduction

Birt–Hogg–Dubé syndrome (BHDS) is an uncommon autosomal dominant hereditary disorder caused by germline mutations in the folliculin (*FLCN*) gene, which is located on chromosome 17p11.2 (1, 2). First described by Birt, Hogg, and Dubé in 1977, and later associated with the *FLCN* gene by Nickerson et al. in 2002, BHDS is characterized by phenotypic heterogeneity, with major clinical manifestations involving the skin (benign cutaneous fibrofolliculomas (FFs)), kidney (renal tumors), and lung (multiple pulmonary cysts (PCs), with a risk of spontaneous pneumothorax (PTX)) (3, 4). The *FLCN* gene, a known tumor suppressor, encodes the folliculin protein, comprising 14 exons. Pathogenic variants in *FLCN* include frameshift, nonsense, splice-site, and missense mutations, most of which result in the loss of function through the generation of premature termination codons (PTCs) and subsequent nonsense-mediated mRNA decay (NMD) (4, 5). While the precise molecular functions of folliculin remain incompletely understood, emerging evidence has significantly advanced our understanding of its role in cellular signaling. Folliculin interacts with key regulatory pathways, including AMP-activated protein kinase (AMPK) and the mechanistic target of rapamycin (mTOR) signaling cascades (6). Moreover, in complex with folliculin-interacting proteins 1 and 2 (FNIP1/2), folliculin functions as a GTPase-activating protein (GAP) for the RagC and RagD GTPases, promoting the mechanistic target of rapamycin complex 1 (mTORC1) activation in response to amino acid availability (7). Additionally, folliculin regulates the transcription factors TFEB and TFE3 by promoting their phosphorylation by mTORC1: mechanistic target of rapamycin complex 1, thereby preventing their nuclear translocation and activation. Loss of *FLCN* function leads to constitutive activation and nuclear localization of transcription factor EB (TFEB) and TFE3, which drives renal cystogenesis and tumorigenesis (8).

In our study, we conducted a long-term follow-up study of five unrelated Chinese families with genetically confirmed BHDS. All probands and selected family members were recruited from central southern China (Hunan Province) and underwent germline *FLCN* mutational analysis via direct Sanger sequencing. Comprehensive clinical data were collected, and genotype–phenotype correlations were evaluated to explore the relationship between *FLCN* variants and the diverse clinical presentations observed.

## 2 Materials and methods

Five families with BHDS were recruited from the Second Xiangya Hospital of Central South University. This study was approved by the Institutional Review Board of the Second Xiangya Hospital, and written informed consent was obtained from all family members. All participants underwent a comprehensive physical examination and other relevant examinations. Genomic DNA was extracted from a 6-ml peripheral blood sample using the DNeasy Blood & Tissue Kit (Qiagen, Valencia, CA). The polymorphism phenotyping-2 (PolyPhen-2), Sorting Intolerant From Tolerant (SIFT), and Mutation Taster programs were used to analyze the effects of mutations on the function of the proteins. Genetic analysis was performed by Sanger sequencing, amplifying all coding exons (exons 4–14) and flanking intronic sequences of the *FLCN* gene using the previously described primers (9). The SWISS-MODEL tool^1^ was used for structural modeling of mutations due to its accuracy in predicting protein conformation changes (10).

## 3 Results

### 3.1 Clinical data

#### 3.1.1 Family 1 (F1)

**Figure 1:**
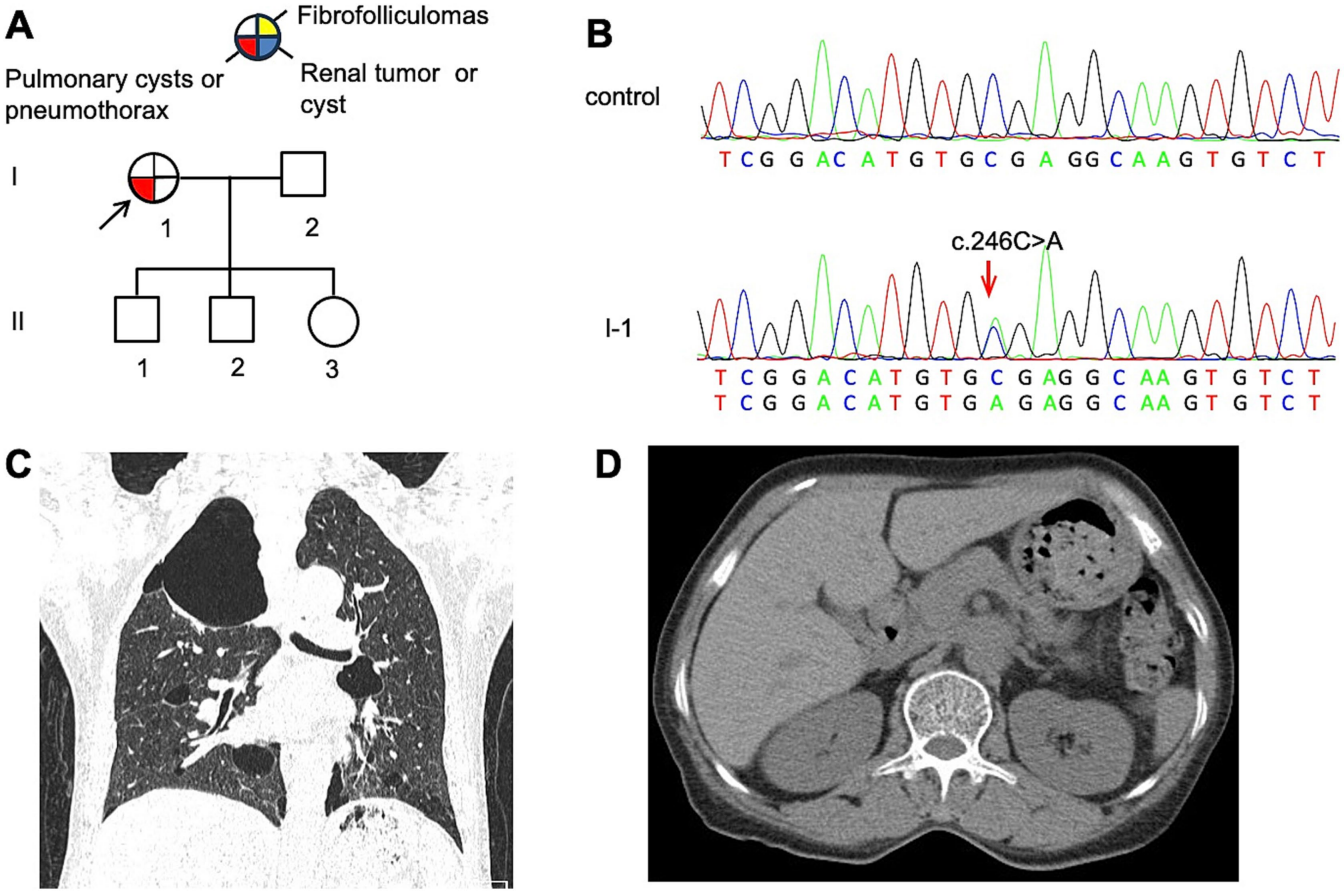
**(A)** Pedigree of the patient’s Family 1. Generations are identified by *Roman* numerals and individuals with *Arabic* numbers. Squares indicate male family members; circles indicate female members; and arrow indicates the proband. **(B)** Sanger sequencing of *FLCN* confirmed a mutation (c.246C > A). **(C)** Lung CT testing results of proband in Family 1. **(D)** Kidney CT testing result for proband in Family 1.

The proband (F1: I-1, Figure 1A), a 57-year-old woman, was admitted to our hospital with a history of chest pain lasting for more than 1 month. High-resolution computed tomography (HRCT) revealed multiple thin-walled PCs of varying sizes, predominantly distributed in the subpleural regions of the lungs (Figure 1C). Despite the absence of typical cutaneous or renal manifestations commonly associated with BHDS (Figure 1D), genetic testing confirmed the presence of a pathogenic variant in *FLCN* (Figure 1B). Her 32-year-old eldest son and 30-year-old second son both underwent comprehensive physical examinations at other hospitals, which revealed no findings suggestive of BHDS. The proband’s 28-year-old youngest daughter reported no symptoms but declined to participate in diagnostic assessments. Genetic testing consent from other family members was not obtained.

#### 3.1.2 Family 2 (F2)

**Figure 2:**
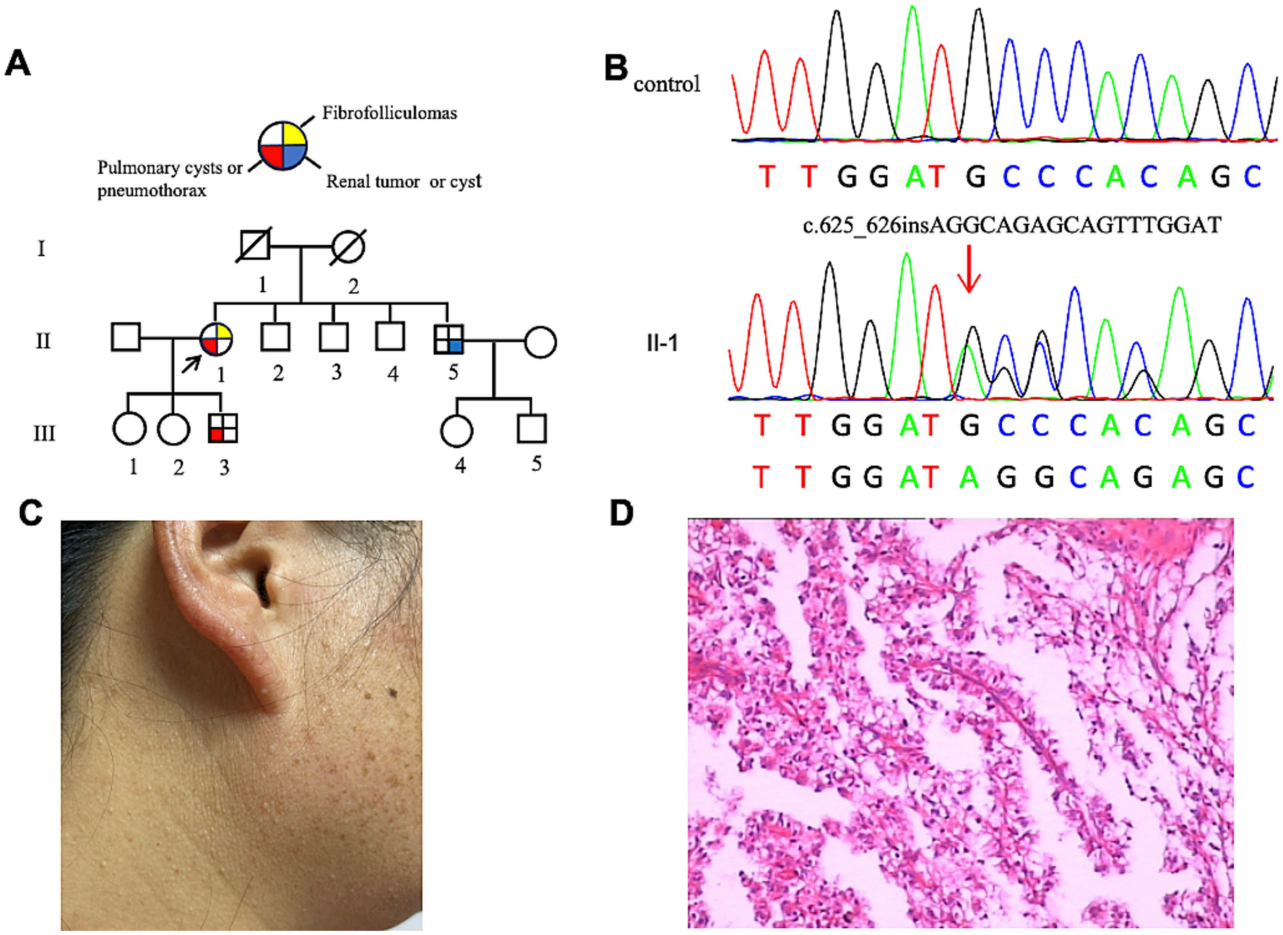
**(A)** Pedigree of the patient’s Family 2. Generations are identified by *Roman* numerals and individuals with *Arabic* numbers. Squares indicate male family members; circles indicate female members; and arrow indicates the proband. **(B)** Sanger sequencing of *FLCN* confirmed a mutation (c.625_626insAGGCAGAGCAGTTTGGAT). **(C)** Fibrofolliculomas in the face and neck of the proband in Family 2. **(D)** Pathological image of the renal tumor tissue in Family 2.

**Figure 3:**
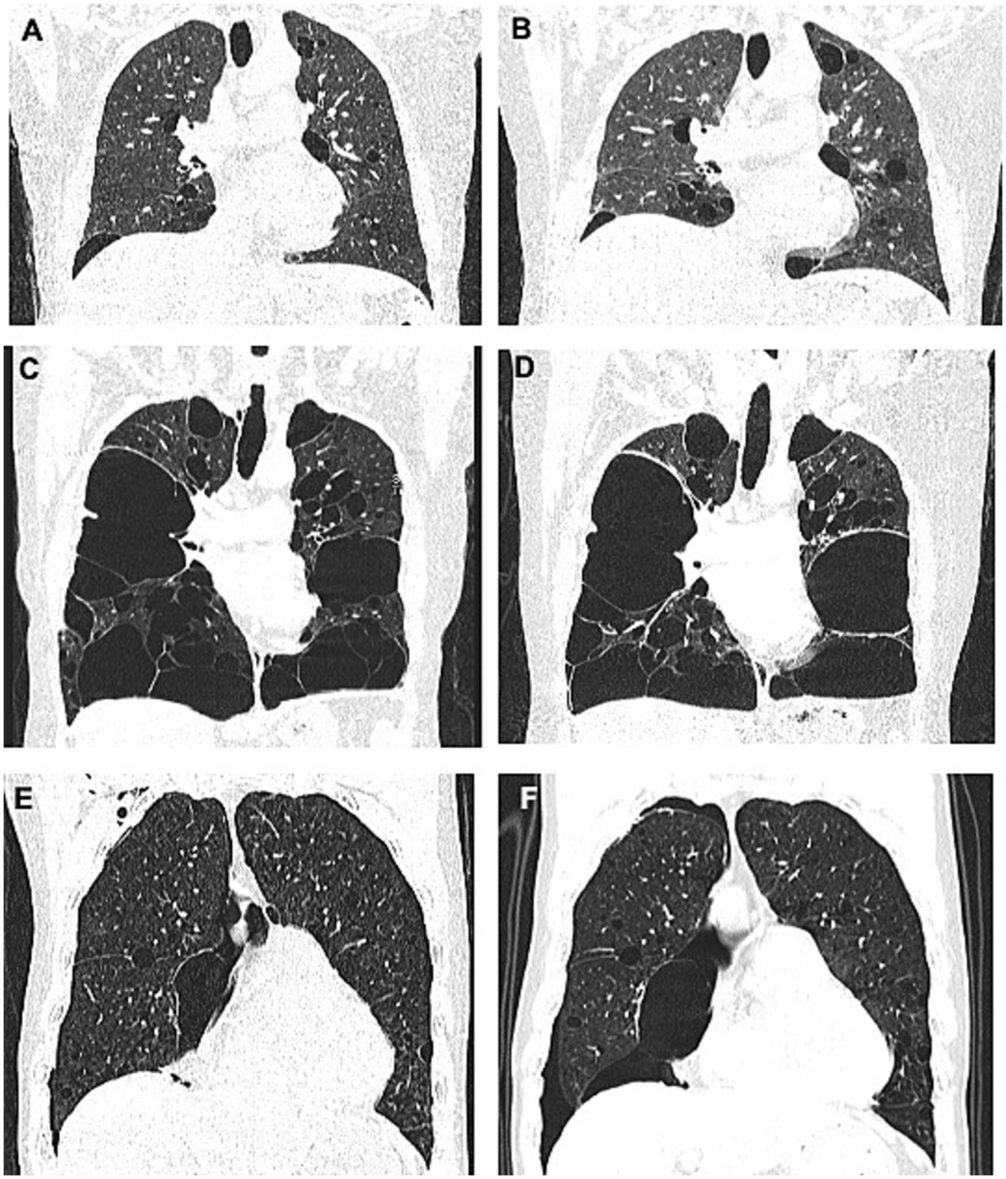
**(A,B)** represent the lung CT results of the proband in Family 2 in May 2019 and January 2024, respectively. **(C,D)** represent the lung CT results of the proband in Family 4 in August 2017 and May 2024, respectively. **(E,F)** represent the lung CT results of the proband in Family 5 in November 2023 and August 2024, respectively.

The proband (F2: II-1, Figure 2A) was a 53-year-old woman who was admitted for chest pain. HRCT revealed multiple PCs of varying sizes, predominantly distributed in the subpleural regions (Figures 3A,B). Abdominal ultrasonography showed no cystic lesions in the kidneys or liver. FFs were observed on her face and neck (Figure 2C). CT imaging of her older brother revealed a left renal mass (45 × 35 × 30 mm) suspicious of malignancy, with no evidence of metastasis. Histopathologic analysis confirmed papillary renal cell carcinoma (Figure 2D). Her youngest son had an episode of primary spontaneous pneumothorax at the age of 27 years, whereas the other two children remained asymptomatic. Given the patient’s multiple skin papules, cystic lesions, and family history of pneumothorax and renal tumors, BHDS was suspected. Genetic testing confirmed the presence of a pathogenic variant in the *FLCN* gene (Figure 2B).

#### 3.1.3 Family 3 (F3)

**Figure 4:**
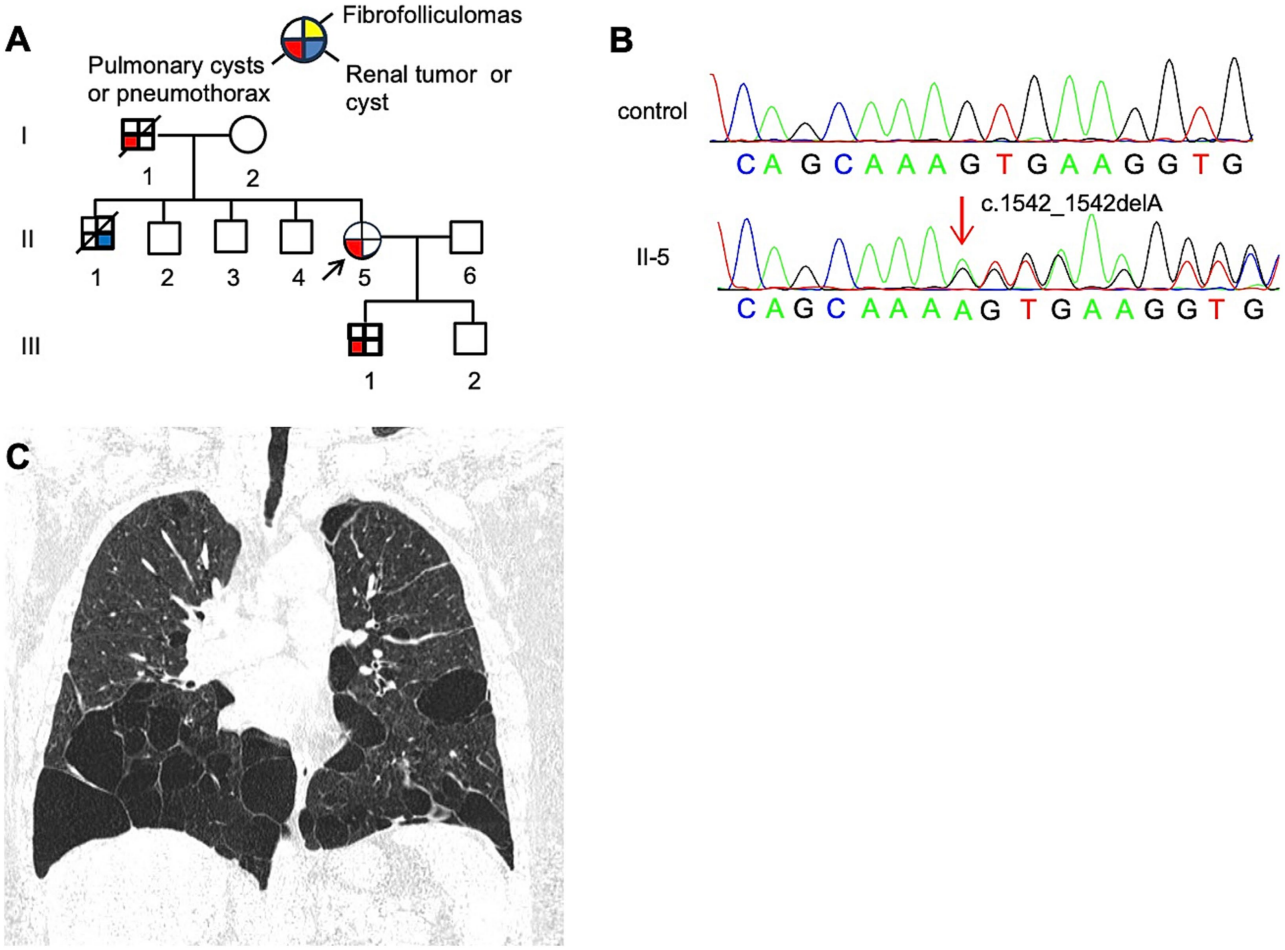
**(A)** Pedigree of the patient’s Family 3. Generations are identified by *Roman* numerals and individuals with *Arabic* numbers. Squares indicate male family members; circles indicate female members; and arrow indicates the proband. **(B)** Sanger sequencing of *FLCN* confirmed a mutation (c.1542_1542delA). **(C)** Lung CT testing result of proband in Family 3.

The proband, a 61-year-old woman (F3: II-5, Figure 4A), presented with dyspnea and was diagnosed with a left-sided pneumothorax based on HRCT (Figure 4C). Ultrasound revealed no renal cysts or tumors, and no skin papules were observed on the face, scalp, neck, or upper chest. Her father (F3: I-1) and older son (F3: III-1) both had a history of pulmonary bullae and spontaneous pneumothorax, which occurred at the ages of 45 and 28, respectively (Figure 4A). According to the proband, her brother (F3: II-1) died of kidney cancer at the age of 60, although medical records were unavailable. Genetic testing was performed only on the proband and her son, both of whom were confirmed to carry a pathogenic variant in the *FLCN* gene (Figure 4B).

#### 3.1.4 Family 4 (F4)

**Figure 5:**
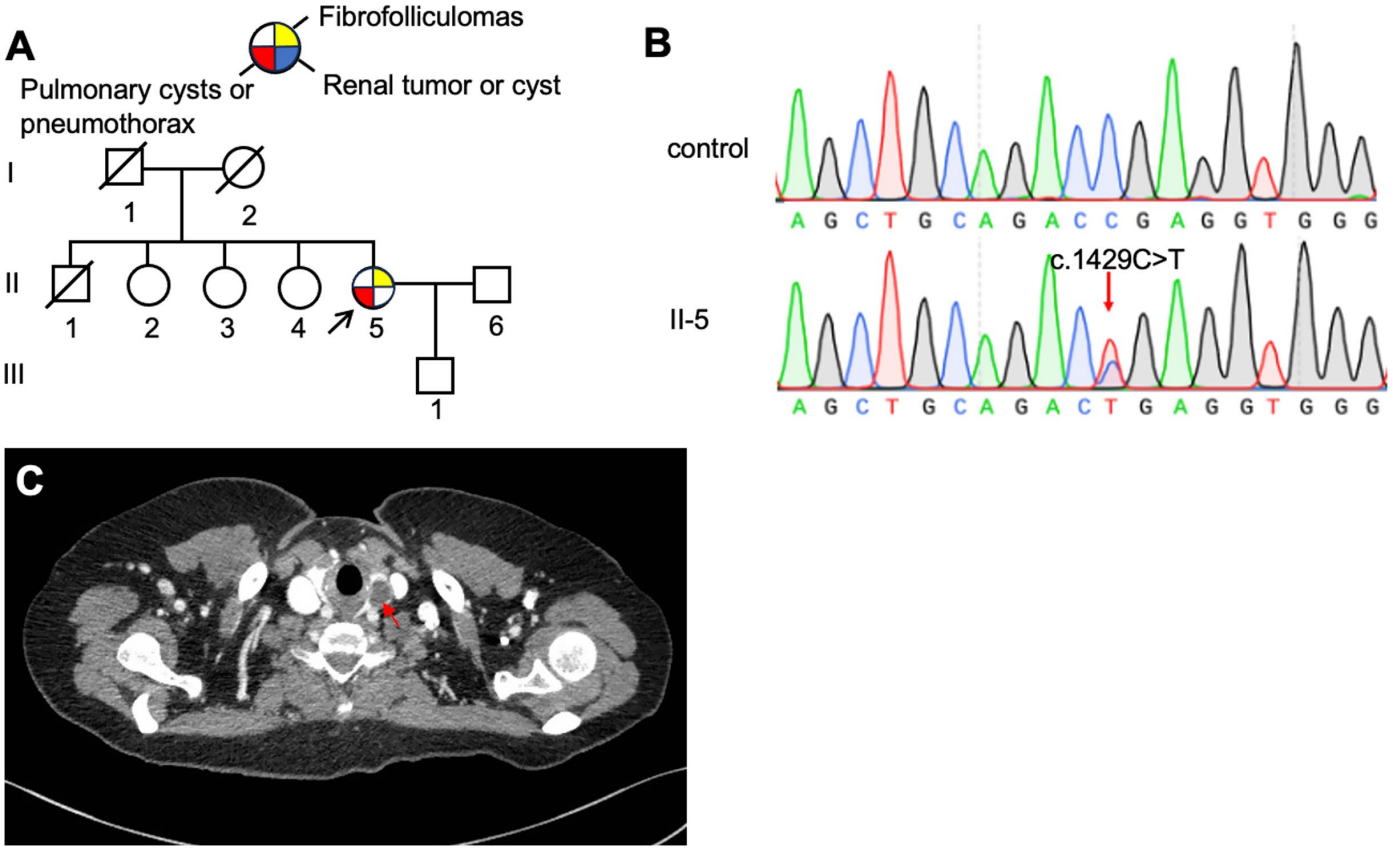
**(A)** Pedigree of the patient’s Family 4. Generations are identified by *Roman* numerals and individuals with *Arabic* numbers. Squares indicate male family members; circles indicate female members; and arrow indicates the proband. **(B)** Sanger sequencing of *FLCN* confirmed a mutation (c.1429C > T). **(C)** Thyroid CT testing result of proband in Family 4, red arrow, thyroid nodule.

The proband (F4: II-5, Figure 5A), a 53-year-old woman, was admitted to the hospital in August 2017 with a 28-year history of recurrent chest tightness and shortness of breath, as well as a recent 20-day episode of right-sided chest pain. HRCT revealed multiple bilateral thin-walled PCs (Figures 3C,D). FFs were observed on the face and neck. During follow-up in May 2024, a left thyroid nodule was detected on HRCT (Figure 5C), while abdominal ultrasonography showed no significant abnormalities. Other family members reported no BHD-related symptoms and declined further clinical evaluation. Genetic testing was performed only on the proband, which confirmed the presence of a pathogenic variant (Figure 5B).

#### 3.1.5 Family 5 (F5)

**Figure 6:**
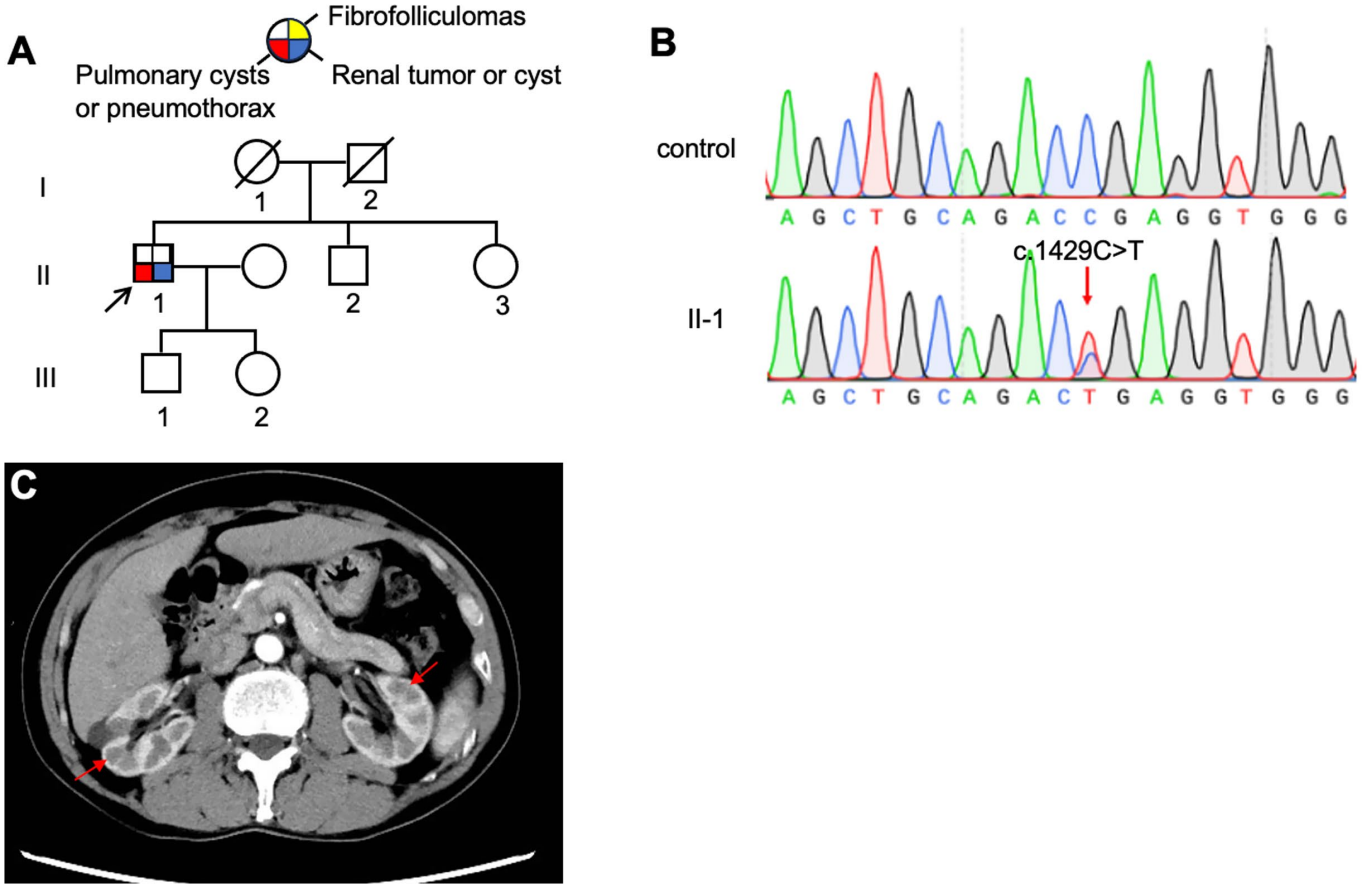
**(A)** Pedigree of the patient’s Family 5. Generations are identified by *Roman* numerals and individuals with *Arabic* numbers. Squares indicate male family members; circles indicate female members; and arrow indicates the proband. **(B)** Sanger sequencing of *FLCN* confirmed a mutation (c.1429C > T). **(C)** Abdominal CT testing result of proband in Family 5.

The proband (F5: II-1, Figure 6A), a 65-year-old man, was admitted to our hospital in October 2023 with sudden dyspnea and right-sided chest pain. HRCT revealed a right-sided pneumothorax and multiple PCs (Figure 3E). He was managed conservatively with tube thoracostomy. Abdominal contrast-enhanced CT revealed bilateral renal cysts (Figure 6C). No cutaneous lesions suggestive of BHDS were observed in any family members. Genetic testing was performed on the proband and four relatives (II-2, II-3, III-1, and III-2), among whom only the proband was found to carry a pathogenic *FLCN* variant (Figure 6B).

### 3.2 Mutation analysis of the *FLCN* gene

Genetic testing confirmed four distinct mutations of the *FLCN* gene in five families (Table 1). All mutations detected were unique, with three being novel. The mutation in each family cosegregated with the disease phenotype and was absent in at least 200 unaffected individuals. A novel (c.246C > A/p.C82*) variant was identified in F1, located in exon 4. This nonsense mutation results in a premature stop codon at position 82. In F2, a novel mutation (c.625_626insAGGCAGAGCAGTTTGGAT/p.C215*) in exon 7 of the *FLCN* gene was detected. This mutation introduces a premature stop codon at codon 215. In F3, a novel nonsense mutation (c.1542_1542delA/p.V515*) in exon 14 of the *FLCN* gene was confirmed. This nonsense mutation led to a premature stop codon at position 515. A previously reported nonsense mutation (c.1429C > T/p.R477*), located in exon 12 of the *FLCN* gene, was identified in both F4 and F5. This nonsense mutation encodes an early termination codon.

**Table 1:** Clinical data and mutation analysis.

Family	Patient No.	Age/Sex	Skin	Lung	Kidney	Others	Site of *FLCN* gene	Nucleotide change	Protein change
F1	I-1	57/F	–	Pulmonary cysts	–	–	exon 4	c.246C > A	p. C82*
F2	II-1	53/F	FFs	Pulmonary cysts	–	–	exon 7	c.625_626insAGGCAGAGCAGTTTGGAT	p. C215*
F2	II-5	43/M	–	–	renal tumors	–	exon 7	c.625_626insAGGCAGAGCAGTTTGGAT	p. C215*
F2	III-1	32/F	–	Pulmonary cysts/PTX	–	–	exon 7	c.625_626insAGGCAGAGCAGTTTGGAT	p. C215*
F3	II-5	61/F	–	Pulmonary cysts/PTX	–	–	exon 14	c.1542_1542delA	p. V515*
F3	III-1	28/M	–	Pulmonary cysts/PTX	–	–	exon 14	c.1542_1542delA	p. V515*
F4	II-5	53/F	FFs	Pulmonary cysts/PTX	–	thyroid nodule	exon 12	c.1429C > T	p. R477*
F5	II-1	65/M	–	Pulmonary cysts/PTX	renal cysts	liver cysts	exon 12	c.1429C > T	p. R477*

FFs, fibrofolliculomas; PTX, pneumothorax.

Mutation Taster, PolyPhen2, FATHMM, and PROVEAN all predicted the four mutations to be “disease-causing,” “probably damaging,” “damaging,” or “deleterious.” Moreover, the newly identified mutation was absent from the dbSNP and Exome Variant Server database.^2^

### 3.3 Disease course and clinical outcomes

Throughout the follow-up period, recurrent pneumothorax was observed in two patients (F4: II-5: 81 months and F5: II-1: 10 months), whereas the three other patients (F1: I-1 at 55 months; F2: II-1 at 56 months; and F3: II-5 at 75 months) remained clinically stable. Follow-up HRCT showed a left thyroid nodule in patient F4: II-5 (Figure 5C), with no significant abnormalities detected on abdominal ultrasonography. Thyroid function tests were within normal limits. HRCT imaging revealed multiple PCs of varying sizes and quantities in three patients (Figure 3), demonstrating a progression in both size and number. No renal tumors were detected in any affected individuals at final follow-up, and the cohort exhibited no mortality throughout the study duration.

### 3.4 Prediction of *FLCN*-mutated protein structure

**Figure 7:**
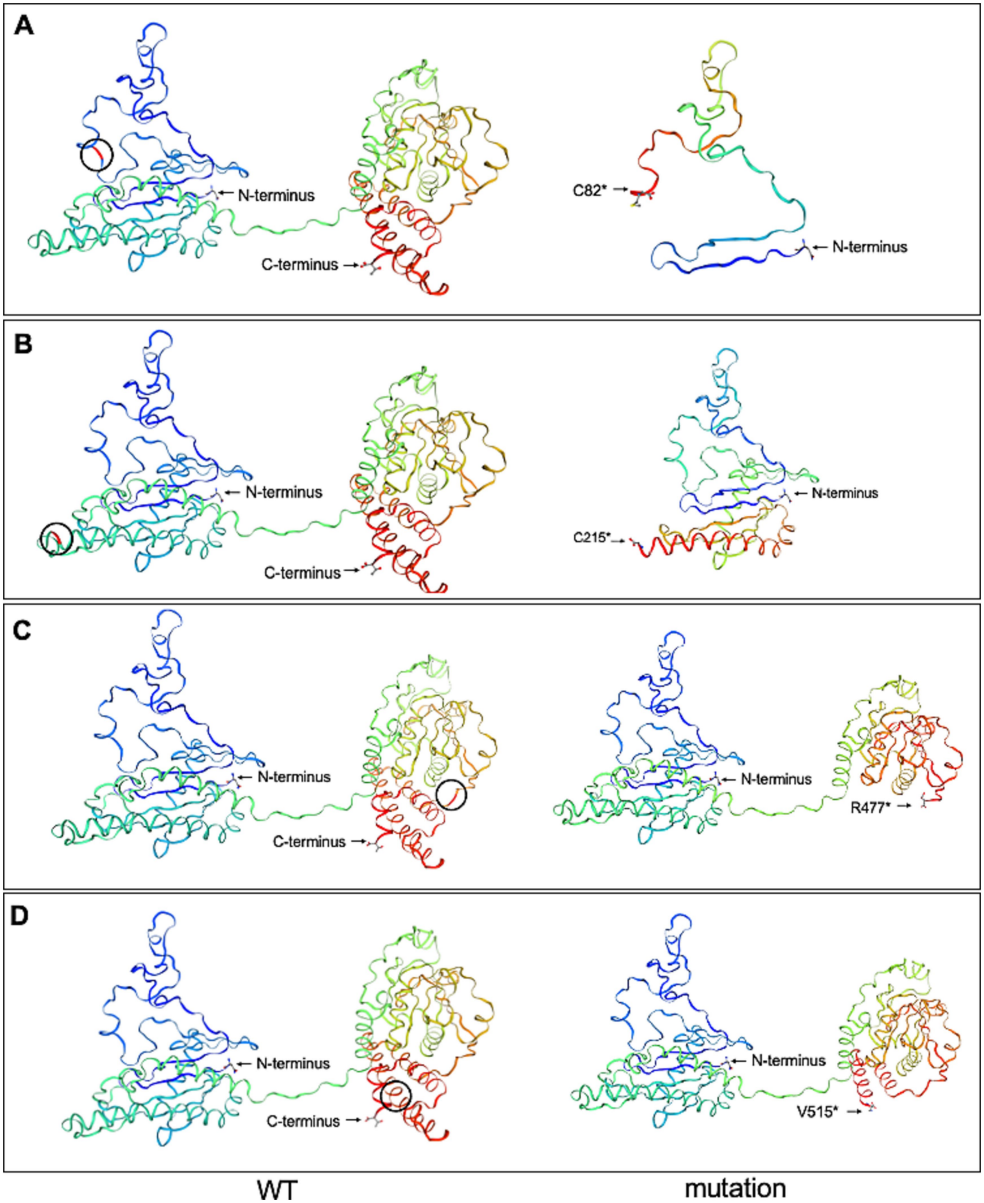
Wild-type *FLCN* (WT) protein structure and the four mutant *FLCN* protein structures were predicted using the SWISS-MODEL online software. **(A)** The mutant *FLCN* (c.246C > A/p. C82*) results in the truncation of the protein at codon 82. **(B)** The mutant *FLCN* (c.625_626insAGGCAGAGCAGTTTGGAT/p. C215*) results in the truncation of the protein at codon 215. **(C)** The mutant *FLCN* (c.1429C > T/p. R477*) results in the truncation of the protein at codon 477. **(D)** The mutant *FLCN* (c.1542_1542delA/p. V515*) results in the truncation of the protein at codon 515. Blue marks the N-terminus (start) and red marks the C-terminus (end) of the protein. Black circles indicate the positions of the changes between WT and mutated proteins.

To investigate the spatial configuration of the *FLCN* mutations, we utilized the SWISS-MODEL online software for structural modeling. Compared to the wild-type *FLCN* protein, the four mutations exhibited significant alterations in their three-dimensional structures, as shown in Figure 7. The four mutations (p.C82*, p.C215*, p.R477*, and p.V515*) introduce premature stop codons in exons 4, 7, 12, and 14, respectively, of the *FLCN* gene. These structural alterations suggest that the mutations produce premature stop codons, leading to the synthesis of truncated proteins that may be intrinsically unstable.

## 4 Discussion

In this study, we identified four pathogenic *FLCN* mutations across five unrelated Chinese families with BHDS, including three novel variants—p.C82*, p.C215*, and p.V515*—and one previously reported mutation, p.R477*. These variants are located in exons 4, 7, 14, and 12, respectively.

Functionally, the loss of *FLCN* impairs its role in regulating autophagy and signaling pathways. The loss of *FLCN* function upregulates mTORC1 activity, leading to impaired autophagy and elevated levels of mTOR pathway downstream effectors, including vascular endothelial growth factor. These molecular changes may contribute to cyst development (11–13). Additionally, Khabibullin et al. demonstrated that *FLCN*-deficient cells exhibit increased cell–cell adhesion forces, which may predispose the lungs to stretch-induced alveolar injury and cyst formation (14–16). All affected individuals in our study consistently exhibited multiple PCs, most of which were irregularly shaped and predominantly located in the basal and peripheral lung regions. Recent structural studies revealed that the N-terminal Longin domain of *FLCN* binds to the Longin domain of FNIP1, while the C-terminal differentially expressed in normal and neoplastic cells (DENN) domain interacts with the DENNc domain of FNIP1. Through FNIP1, *FLCN* associates with RagA, RagC, and the Ragulator complex to regulate mTORC1 activation (17, 18). The four mutations identified in our study introduce PTCs, resulting in truncated folliculin proteins that may lack one or both of the functional domains. Such truncations are predicted to destabilize the protein and disrupt critical interactions with FNIP1 and the Rag GTPases, thereby impairing mTORC1 activation and lysosomal signaling. These molecular disruptions may explain the observed phenotypic variability among patients, including differences in pulmonary, renal, and cutaneous involvement.

Notably, the p.R477* mutation, located near the 3′ end of exon 12, was detected in two families (F4 and F5). Previous reports of p.R477* have described cases with cutaneous FFs (19), lung cysts and pneumothorax without renal involvement (20), isolated renal cell carcinoma (21), and mixed renal/pulmonary phenotypes (22). Intriguingly, bioinformatics analysis suggests that c.1429C > T may not only introduce a stop codon but also disrupt *FLCN* splicing, which could further contribute to functional loss (23). In our study, two individuals (F5: II-5 and F6: II-1) carrying this variant presented with bilateral lung cysts, with one of them (F5: II-5) later developed a thyroid nodule during follow-up. Although thyroid lesions are not considered hallmark features of BHDS, a cohort study has suggested a higher prevalence of thyroid nodules in BHDS patients (24). However, given the lack of a control group, causality remains unconfirmed, and age-related factors must be considered.

In our study, renal involvement was relatively uncommon. Only one patient (F2: II-5) was diagnosed with renal cell carcinoma, and another (F5: II-1) had renal and liver cysts. This low frequency may be attributed to referral bias, as most participants were recruited from a respiratory clinic, and some relatives declined abdominal imaging. Nonetheless, all probands were advised to undergo annual renal surveillance with ultrasound or CT scans due to the known risk of renal malignancy in BHDS.

Interestingly, only two cases of typical FFs were identified (F2: II-1, F4: II-5), further supporting previous observations that Asian BHDS patients may have a lower prevalence of cutaneous manifestations compared to Caucasian cohorts. For example, while Toro et al. (25) reported FFs in 90% of patients and renal tumors in 34%, studies from China, Japan, and the Republic of Korea have consistently shown higher rates of pulmonary involvement and lower rates of skin and renal lesions (26–29). This geographical and ethnic variability underscores the need for region-specific genotype–phenotype studies.

Longitudinal data from one patient (F2: II-1) showed progressive enlargement and an increased number of pulmonary cysts over 56 months, suggesting a possible correlation between novel truncating variants and disease progression. Mutations in exon 12 (25), such as p.R477*, have previously been associated with more severe pulmonary phenotypes, including increased cyst burden and pneumothorax risk, which is consistent with our findings.

Overall, our study expands the mutational spectrum of *FLCN* in the Chinese population by identifying three novel truncating mutations in exons 4, 7, and 14. These findings reinforce the pathogenic significance of truncating variants in BHDS and highlight the clinical and genetic heterogeneity of the disease. Molecular characterization of *FLCN* mutations not only facilitates early diagnosis and risk stratification but also provides valuable information for genetic counseling and family screening. Future large-scale studies integrating genotype, expression profiling, and longitudinal clinical outcomes are warranted to deepen our understanding of BHDS pathogenesis and its diverse phenotypic spectrum.

## Data Availability

The original contributions presented in the study are included in the article/Supplementary material, further inquiries can be directed to the corresponding authors.

## Footnotes

^1^ https://swissmodel.expasy.org/interactive

^2^ http://evs.gs.washington.edu/EVS/
