# Supplementary material for: Clinical and genetic investigations of five Chinese families with Birt–Hogg–Dubé syndrome: a long-term follow-up study
Source: Front Med (Lausanne). 2025 Sep 8;12:1613154. doi: 10.3389/fmed.2025.1613154 (PMC12452094; doi:10.3389/fmed.2025.1613154)
Supplement: Supplementary file 1 [file Supplementary_file_1.docx]

Summary Table

| Family | PatientNo. | Age/Sex | FLCN Variant (NM_144997) | Clinical presentation | Affected Relatives Tested | Follow-up Duration |
| --- | --- | --- | --- | --- | --- | --- |
| F1 | I-1 | 57/F | c.246C>A (p.C82*) | no skin lesions, PC, no renal tumor | No | 55 months |
| F2 | II-1 | 53/F | c.625_626insAGGCAGAGCAGTTTGGAT (p.C215*) | FFs, PC, PTX, renal tumor | Yes (II-5, III-1) | 56 months |
| F3 | II-5 | 61/F | c.1542_1542delA (p.V515*) | no skin lesions, PC, PTX | Yes (III-1, III-2) | 75 months |
| F4 | II-5 | 53/F | c.1429C>T(p.R477*) | FFs, PC, PTX, thyroid nodule, no renal tumor | No | 81 months |
| F5 | II-1 | 65/M | c.1429C>T(p.R477*) | no skin lesions, PC, PTX, renal cysts | Yes (II-2, II-3, III-1, III-2) | 10 months |

FFs: fibrofolliculomas; PTX: pneumothorax.
